# Mapping individual voxel-wise morphological connectivity using wavelet transform of voxel-based morphology

**DOI:** 10.1371/journal.pone.0201243

**Published:** 2018-07-24

**Authors:** Xun-Heng Wang, Yun Jiao, Lihua Li

**Affiliations:** 1 College of Life Information Science and Instrument Engineering, Hangzhou Dianzi University, Hangzhou, China; 2 Jiangsu Key Laboratory of Molecular and Functional Imaging, Department of Radiology, Zhongda Hospital, Medical School of Southeast University, Nanjing, China; University at Buffalo, UNITED STATES

## Abstract

Mapping individual brain networks has drawn significant research interest in recent years. Most individual brain networks developed to date have been based on fMRI or diffusion MRI. Given recent concerns regarding confounding artifacts, various preprocessing steps are generally included in functional or structural brain networks. Notably, voxel-based morphometry (VBM) derived from anatomical MRI exhibits high signal-to-noise ratios and has been applied to individual interregional morphological networks. To the best of our knowledge, individual voxel-wise morphological networks remain unexplored. The goal of this research is twofold: to build novel metrics for individual voxel-wise morphological networks and to test the reliability of the proposed morphological connectivity. To this end, anatomical scans of a cohort of healthy subjects were obtained from a public database. The anatomical datasets were preprocessed and normalized to the standard brain space. For each individual, wavelet-transform was applied on the VBM measures to obtain voxel-wise hierarchical features. The voxel-wise morphological connectivity was computed based on the wavelet features. Reliable brain hubs were detected by the z-scores of degree centrality. High reliability was discovered by test-retest analysis. No effects of wavelet scale, network threshold or network type were found on hubs of group-level degree centrality. However, significant effects of wavelet scale, network threshold and network type were found on individual-level degree centrality. Significant effects of network threshold and network type were found on reliability of degree centrality. The results suggested that the voxel-wise morphological connectivity was reliable and exhibited a hub structure. Moreover, the voxel-wise morphological connectivity could reflect individual differences. In summary, individual voxel-wise wavelet-based features can probe morphological connectivity and may be beneficial for investigating the brain morphological connectomes.

## Introduction

Voxel-based morphometry (VBM) is a neuroimaging tool that can probe local differences of brain anatomy and has been applied in statistical analysis [1, 2] and data mining [3–5]. The brain is a complex network and contains high-order cortical information, although the local features of the brain morphology do not reflect the brain topology. Individual brain topology has been widely investigated by diffusion MRI and functional MRI [6, 7]. Meaningful network topology properties (i.e., hubs, scale-free, small-worldness) have been found in the human brain [8–10]. Given recent concerns regarding confounding artifacts, various preprocessing steps are generally implemented in functional or structural brain networks [11, 12]. Notably, VBM features that exhibit high signal-to-noise ratios can be derived from anatomical MRI with well-established preprocessing procedures. Hence, investigating the individual morphological relationships based on VBM features may be beneficial for studies of the brain connectome.

Mapping individual morphological relationships is a challenging task based on local features of an anatomical volume. Interregional image density distribution-based Kullback-Leibler (KL) divergence has been recently applied to measure individual morphological connectivity [13]. The interregional KL similarity was shown to be reliable [14] and related to brain maturation [15]. Hence, it is feasible to map individual morphological connectivity using interregional measures. Although kernel density estimation exhibited good performance across networks of different resolutions [14], the different probability distribution functions might influence the morphological connectivity [15]. Thus far, biological explanations of density distribution-based similarity remain unclear [13]. Moreover, the aforementioned studies relied on regional statistics, and therefore, the results cannot be extended to investigations of individual voxel-wise morphological connectivity.

Mapping inter-voxel morphological relationships at the single-subject level remains largely underexplored, as there is a lack of local features in a single voxel of an anatomical volume. Notably, voxel patch-based similarity has been applied to extract individual brain networks from structural MRI scans [16], which has shed light on voxel-wise anatomical networks. To obtain morphological relationships, the brain was divided into thousands of cubes, which served as network nodes. The inter-cube morphological relationship was defined as the correlation between the two cubes of 27 voxels [16]. However, the cubes could not represent the complex structure of the brain, which exhibited variability across subjects. Moreover, the voxel patch could only capture the local features within a cube and did not reflect the global features of functionally or structurally homogeneous brain regions [16]. Thus, the cubes of voxel patch features should be replaced by a vector of hierarchical features that contains both local and global features in a single voxel.

Wavelet transform has been applied in brain morphometry studies [17]. The wavelet transform is a multiscale analysis method that can transform the energy of a signal into a multi-resolution hierarchical organization. Hence, wavelet transform can capture both local and global features of an anatomical MRI dataset. Wavelet-based morphometry (WBM) is a novel method that can probe structural morphometric differences [17]. Moreover, the wavelet transform is a multivariate method that has been applied in diagnostic models of brain disorders [18]. Diagnostic models based on wavelet transform exhibit higher sensitivity and specificity than conventional methods [18]. Therefore, wavelet transform could be beneficial for the analysis of anatomical MRI. Considering the hierarchical organization of wavelet features, investigating the voxel-wise relationships using wavelet transform is a straightforward approach.

In this paper, we aimed to map the individual voxel-wise morphological connectivity based on anatomical MRI and to test the reliability and variability of the voxel-wise morphological connectivity. To this end, a cohort of adult participants was obtained from a publicly available MRI database. The method section describes the application of the wavelet transform to the VBM data to obtain multi-resolution features for a single volume. Wavelet-based similarity was applied as the voxel-wise morphological connectivity. In the result section, the reliability and variability of the voxel-wise morphological connectivity were evaluated using statistical analysis. In the discussion section, we summarize the reliability and variability of the voxel-wise morphological networks.

## Methods

### Subjects and MRI protocols

Twenty-one healthy subjects (11 males and 10 females, 22–61 years old) without neurological history were recruited at Vanderbilt University, USA [19]. For each participant, one structural MRI dataset was obtained using a 3T Philips Achieva MR scanner. This study was approved by the institutional review boards of Vanderbilt University, USA. Signed informed consent was provided by each participant. Each subject was scanned twice with a one-hour break between scans. All scans were completed within 2 weeks. The structural MRI dataset was acquired with a magnetization-prepared rapid acquisition gradient echo (MPRAGE) sequence (repetition time = 6.7 ms, echo time = 3.1 ms, inversion time = 842 ms, 1 mm×1 mm×1.2 mm, 6 minutes). The datasets are publicly available without restrictions for academic usage (https://www.nitrc.org/projects/multimodal/).

### Data preprocessing

The structural MRI datasets were first reoriented to the standard space, automatic cropped, bias field corrected, and skull-stripped to exclude non-brain tissues. The brain images were then segmented into gray matter, white matter, and cerebrospinal fluid. Third, the original and segmented images were spatially normalized to the standard brain space. Fourth, the spatial normalized gray matter image was multiplied by the Jacobian of the warp field. Finally, the modulated gray matter image was obtained as the VBM features. The spatial normalization procedure was carried out using the fsl_anat command. The VBM procedure was carried out using the fslvbm command.

### Wavelet transform

The wavelet transform was applied to extract local and global features for the VBM volumes, resulting in hierarchical features for each single voxel. Thus, the wavelet transform could provide richer information than the conventional VBM method. A wavelet can be defined as a small wave-like oscillation that can be used to decompose a time-domain or spatial-domain signal into different scales [18]. The wavelet transform first decomposes the original 3D images into sub-bands at different spatial scales and then adaptively concentrates the original 3D spatial information into a sets of wavelet coefficients [17]. Here, the multi-resolution analysis was carried out through a discrete orthogonal wavelet transform, which adaptively decomposed the images at different spatial-scale multi-frequency bands based on a small series of wavelet coefficients [17]. The discrete orthogonal wavelet transform was successfully applied in wavelet-based morphometry. For each subject, discrete orthogonal wavelet transform-based feature extraction was conducted using the following steps: 1) The wavedec3 function in MATLAB is used for multilevel 3D wavelet decomposition of the VBM volume; 2) The coefficients of the wavelet components are obtained and saved; 3) The waverec3 function in MATLAB is used to obtain the 3D wavelet reconstruction of approximations and details; 4) Each wavelet component is normalized according to the z-scores; 5) The approximations and details are concentrated into a 4D volume. Here, the approximations represent low-pass components of the wavelet transform, while the details indicate high-pass components of the wavelet transform. Specifically, the approximations denote global features that change over the long space, while the details denote local features that change over the short space.

### Voxel-wise morphological connectivity

The nodes and edges of brain networks should be defined before computing voxel-wise morphological relationships. The locations of nodes were defined using the first 90 brain regions in the automated anatomical labeling (AAL) atlas, resulting in 159841 nodes in the 2 mm×2 mm×2 mm 3D space. Given a voxel-wise morphological network, the morphological connectivity (edge) between two voxels (nodes) can be defined by the Pearson correlation coefficient of pairwise wavelet feature vectors in brain voxels.

$$r(w_{1}{,w}_{2})=\frac{cov(w_{1}{,w}_{2})}{\delta_{w_{1}}\delta_{w_{2}}}$$

Here, w denotes the wavelet feature vector within a voxel, cov denotes covariance, and δ denotes the standard deviation of the wavelet feature vector. For a wavelet scale of n, the length of the wavelet feature vector is 2*n.

For a binarized graph, the links are defined as:

$$a_{ij}=\left\{ \begin{array}{c} 1,r_{ij}\geq r_{0}\\ 0,r_{ij}<r_{0}. \end{array}\right.$$

For a weighted graph, the links are defined as:

$$a_{ij}=\left\{ \begin{array}{c} r_{ij},r_{ij}\geq r_{0}\\ 0,r_{ij}<r_{0}. \end{array}\right.$$

Here, i and j denote the two voxels, and *r*_0_ denotes the threshold for a graph [20]. The thresholds *r*_0_ for morphological connectivity were set to 0.5, 0.6, 0.7, 0.8 and 0.9 for sparsity.

**Fig 1:**
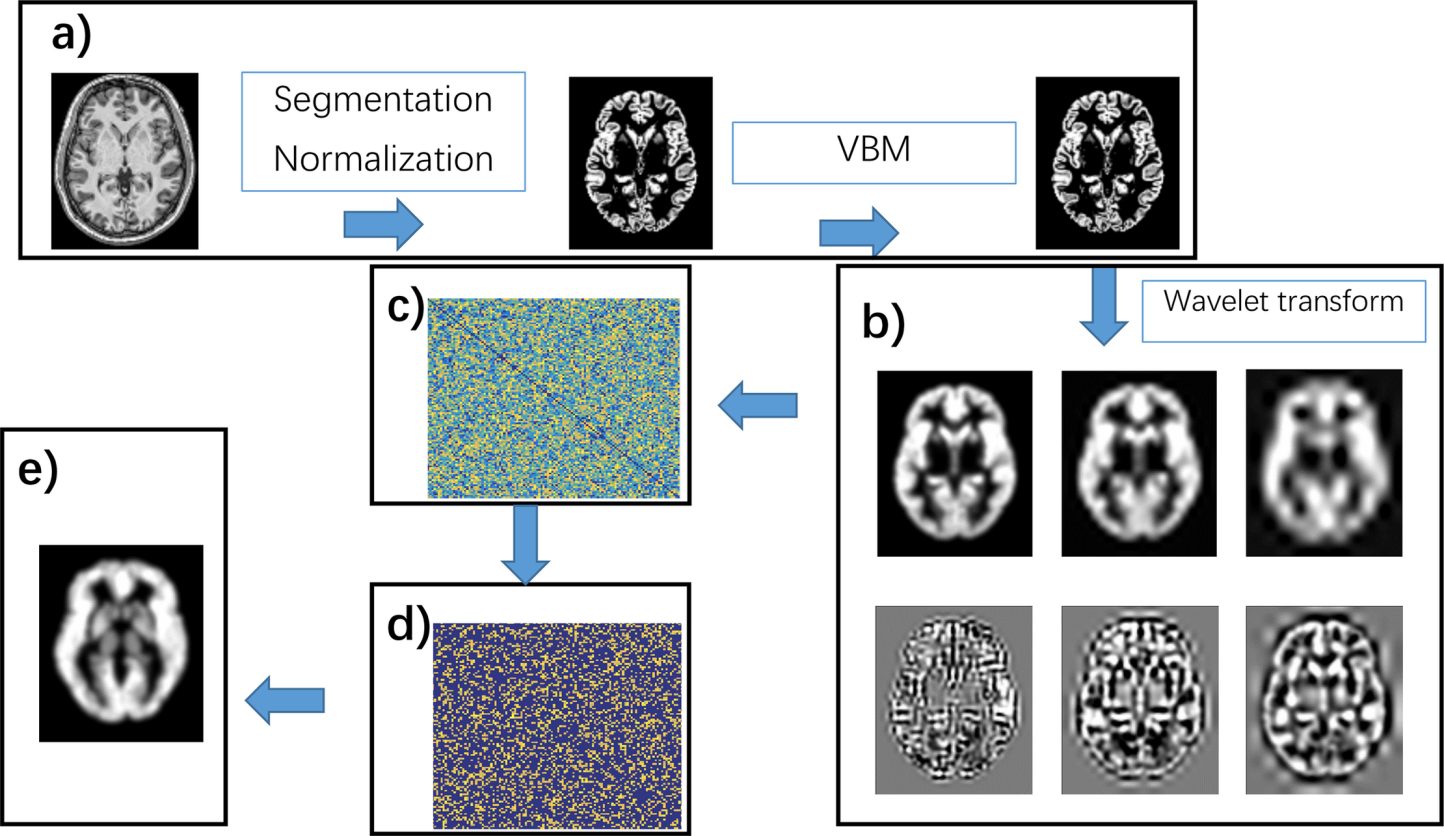
Pipeline for individual voxel-wise morphological connectivity. The pipeline contains the following steps: a) VBM transform; b) wavelet transform; c) Pearson correlation; d) thresholding; e) degree centrality. In pipeline b, the top three images represent the details of the wavelet transform, and the bottom three images represent the approximations of the wavelet transform. The brain mask was constructed by skull-stripping the template image in FMRIB Software Library (FSL).

Given a wavelet-transformed 4D VBM volume for each individual, voxel-wise morphological relationships were computed by the following steps: 1) The voxel-wise wavelet features are obtained; 2) The voxel-wise features are masked using the AAL atlas; 3) the Pearson correlation coefficients of pairwise wavelet features between the two voxels are derived; 4) The voxel-wise morphological connectivity is obtained; 5) Thresholds (i.e.,0.5, 0.6, 0.7, 0.8, 0.9) are applied to the voxel-wise morphological connectivity; 6) The voxel-wise nodal degree of morphological connectivity is obtained. The pipeline to obtain individual voxel-wise morphological connectivity is illustrated in Fig 1. The scripts can be obtained from the Neuroimaging Informatics Tools and Resources Clearinghouse (NITRC) website (https://www.nitrc.org/projects/mcwt/).

### Degree of centrality

Centralities are the most common graph measures for nodal estimators (i.e., degree centrality, betweenness centrality, eigenvector centrality) [7, 20–22]. Degree centrality is one of the most common measures of centrality and is defined as the number of links (neighbors) connected to a node [7]. In this paper, degree centrality was applied to investigate the nodal properties of the morphological networks. Degree centrality was calculated using a publicly available script in the Data Processing Assistant for Resting-State fMRI (DPARSF) toolbox [23].

Both binarized and weighted graphs were analyzed through thresholding the symmetrical matrix of morphological connectivity [20]. Degree centrality for binarized networks was derived by computing the sum of the number of edges within the binarized graph. Degree centrality for weighted networks was derived by computing the sum of the values of the weighted edges within the weighted graph. Before statistical analysis, the individual maps of degree centrality were spatially smoothed using the fslmaths function with a Gaussian kernel (sigma = 3 mm). To analyze the effects of wavelet scale (i.e., 3, 4, 5), network threshold (i.e., 0.5, 0.6, 0.7, 0.8, 0.9), and network type (i.e., binarized or weighted), the raw scores of degree centrality were transformed into z-scores using the following formula:

$Z_{i}=\frac{D_{i}-mean(D)}{std(D)}$, where *D_i_* indicates the *i*th voxel-wise degree centrality, D indicates the whole-brain voxel-wise degree centrality, and std indicates the standard deviation.

### Hub detection

Hub voxels contain more connections than other voxels within a voxel-wise network. To detect the hubs of the voxel-wise networks, the raw scores of degree centrality were first transformed into z-scores. Then, group-level z-scores of degree centrality were obtained by averaging the individual degree centrality with different effects (i.e., wavelet scales, network thresholds, types of network). Finally, for each effect factor, the hub voxels were indicated by those with a z-score >1, according to previous studies [24–26].

### Test-retest analysis

A test-retest analysis was applied to investigate the reliability of the proposed voxel-wise morphological network. The test-retest reliability was analyzed via the intraclass correlation coefficients (ICCs) [27, 28]. In this paper, the ICCs were computed based on a random effects model and were given by the ratio of the intersubject variance to the total variance. According to previous studies [29], the ICCs were partitioned into five levels: excellent reliability (ICC>0.8), high reliability (0.6<ICC<0.79), moderate reliability (0.4<ICC<0.59), fair reliability (0.2<ICC<0.39), and poor reliability (ICC<0.2).

### Statistical analysis

To analyze the effects of wavelet scale (i.e., 3, 4, 5), network threshold (i.e., 0.5, 0.6, 0.7, 0.8, 0.9), and network type (i.e., binarized or weighted) on the network hubs and network reliability, three-way Analysis of Variance (ANOVA) was performed on the group-level degree centrality or reliability maps using the following formula:

Y∼A+B+C

Here, Y denotes group-level degree centrality or reliability maps, and A, B, and C denote wavelet scales, network thresholds, and network types, respectively.

To analyze the effects of wavelet scale, network threshold, and network type on individual differences in degree centrality, repeated-measures ANOVA was performed on the individual-level degree centrality maps using the following formula:

DC∼A*B*C+Error(subject/(A*B*C))

Here, DC denotes the individual-level degree centrality, and A, B, and C denote wavelet scales, network thresholds, and network types, respectively. The results were corrected by the false discovery rate (FDR).

## Results

### Group-level degree centrality of voxel-wise networks

**Fig 2:**
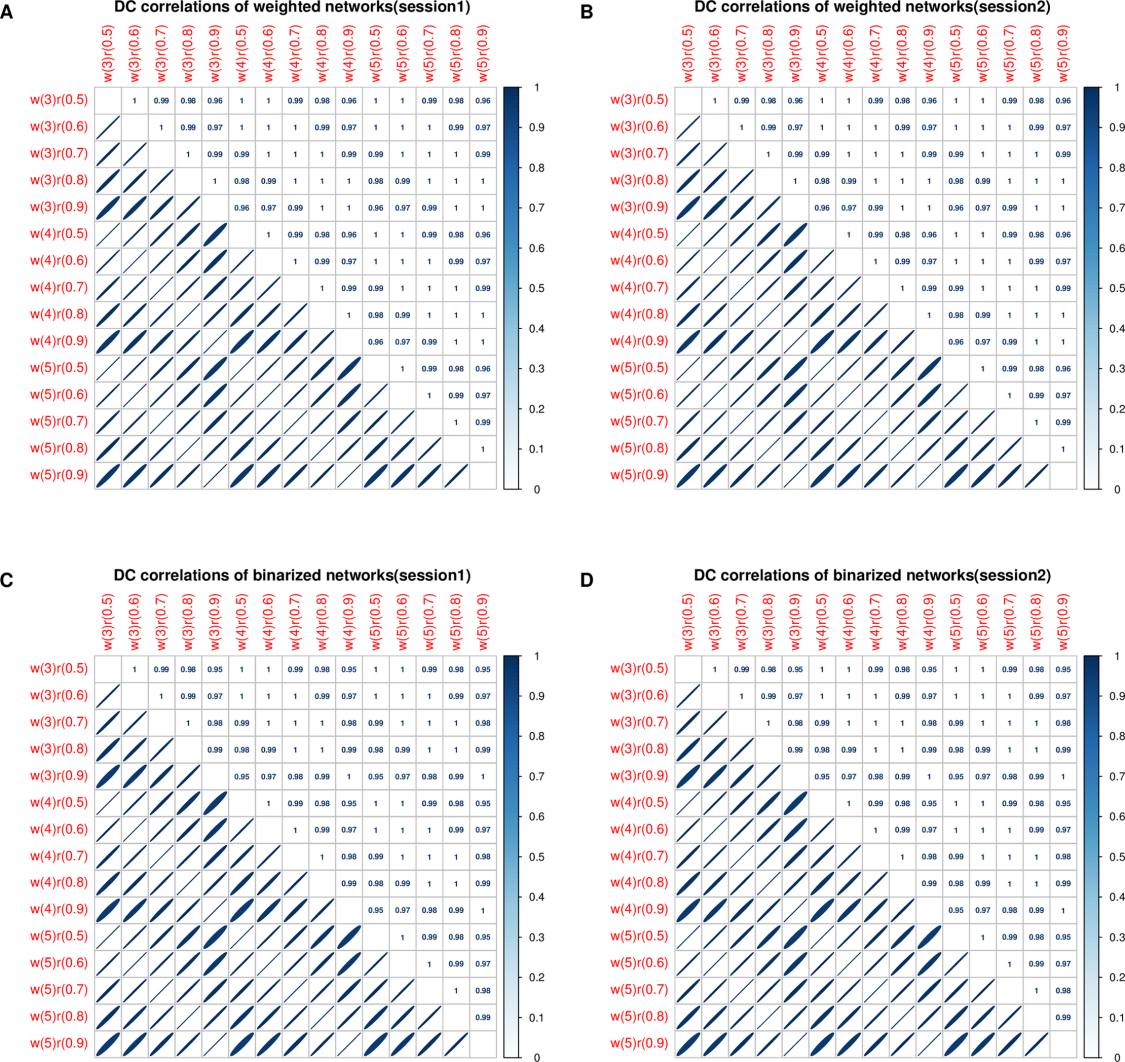
Degree centrality correlations among different networks. Subfigure A denotes the degree centrality correlations of weighted networks in session 1. Subfigure B denotes the degree centrality correlations of weighted networks in session 2. Subfigure C denotes the degree centrality correlations of binarized networks in session 1. Subfigure D denotes the degree centrality correlations of binarized networks in session 2. w indicates wavelet scale, and r indicates the network threshold, e.g., w(3)r(0.5) denotes a brain network with a wavelet scale of 3 and a threshold of 0.5.

Table 1 shows the network sparsity based on different network thresholds. Table 2 and Table 3 present the results of the three-way ANOVA on group-level degree centrality among the different kinds of networks. There are no effects of wavelet scale, network threshold or network type on group-level degree centrality. Fig 2 shows the correlations of group-level degree centrality among different kinds of networks. The maps of degree centrality are significantly correlated with each other, and most of the correlation coefficients are close to 1. The correlation coefficients are also reliable with different sessions. The group-level degree centrality exhibits reliable spatial patterns across different wavelet scales, network thresholds and network types.

**Table 1:** Network sparsity based on different network thresholds.

	r = 0.5	r = 0.6	r = 0.7	r = 0.8	r = 0.9
scale 3	27.14% ± 0.61%	22.99% ± 0.6%	18.45% ± 0.56%	13.34% ± 0.48%	7.36% ± 0.32%
scale 4	23.67% ± 0.46%	18.97% ± 0.44%	14.05% ± 0.39%	8.93% ± 0.3%	3.78% ± 0.16%
scale 5	22.13% ± 0.45%	17.16% ± 0.42%	12.12% ± 0.37%	7.12% ± 0.38%	2.56% ± 0.13%

**Table 2:** ANOVA of degree centrality for session 1.

	sum square	mean square	F value	p-value
wavelet scale	0	0	0	1
threshold	0	0	0	1
network type	0	0	0	1

**Table 3:** ANOVA of degree centrality for session 2.

	sum square	mean square	F value	p-value
wavelet scale	0	0	0	1
threshold	0	0	0	1
network type	0	0	0	1

### The hub brain regions in the voxel-wise networks

**Fig 3:**
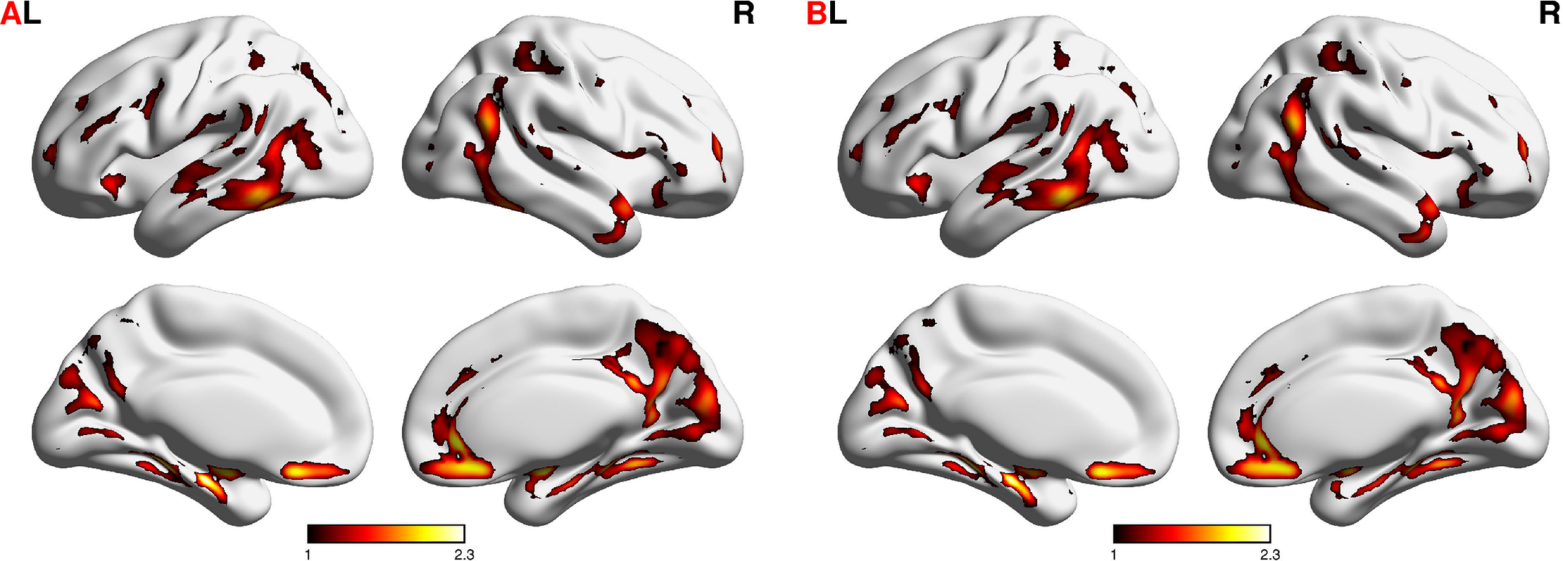
Brain hubs across different networks. Subfigure A denotes brain hubs in session 1. Subfigure B denotes brain hubs in session 2. The brain hubs are indicated by a z-score >1.

Fig 3 shows the brain hubs (i.e., precuneus, cingulate gyrus, medial frontal gyrus, superior frontal gyrus, superior temporal gyrus, middle temporal gyrus, middle occipital gyrus, cuneus, insula, hippocampus, and amygdala) of the voxel-wise networks. The brain hub regions in the voxel-wise networks exhibit reliable spatial patterns across the two scan sessions. Fig 3A shows the brain hubs based on the average networks of session 1, and Fig 3B shows the brain hubs based on the average networks of session 2. The brain hubs are indicated by a z-score >1, which represents the cores of connections. The average percentages of high degree centrality (i.e., z-score >1) for session 1 and session 2 are 14.16% and 14.25%, respectively. The 10 brain hub regions of session 1 are listed in Table 4, and the 11 brain hub regions of session 2 are listed in Table 5. The sizes and locations of the hub regions are comparable between the two sessions.

**Table 4:** Brain hubs in session 1.

cluster^a^	L/R^b^	BA^c^	MNI (x,y,z)^d^	K^e^	Peak value^f^
1	L/R	6, 7,9, 10,11, 13, 17, 19, 21, 22, 23, 24, 30, 31, 32, 37,40,47	(2, 40, -8)	17114	3.21
2	L	13, 20, 21, 22, 37, 40, 41	(-46, 6, -2)	4433	2.02
3	R	10	(32, 56, 8)	246	1.84
4	R	19	(36, -88, 2)	54	1.32
5	L	10	(-30, 56, 6)	160	1.78
6	L	9, 46	(-46, 10, 30)	430	1.71
7	R	9, 10	(32, 42, 30)	67	1.32
8	L	8, 9, 10	(-30, 40, 32)	91	1.25
9	R	8	(30, 26, 46)	12	1.06
10	R	6	(36, -4, 58)	22	1.08

^a^ Reported by xjView.

^b^ Left or right cerebral hemisphere.

^c^ Brodmann areas.

^d^ Montreal Neurological Institute coordinates for the peak voxel.

^e^ Number of voxels in each cluster.

^f^ Peak value of degree centrality.

**Table 5:** Brain hubs in session 2.

cluster^a^	L/R^b^	BA^c^	MNI (x,y,z)^d^	K^e^	Peak value^f^
1	L/R	6, 7,9, 10,11, 13, 17, 19, 21, 22, 23, 24, 30, 31, 32, 37,40,47	(2, 40, -8)	17196	3.2
2	L	13, 20, 21, 22, 37, 40, 41	(-38, 22, -4)	4518	2.02
3	R	10	(34, 54, 10)	242	1.81
4	R	19	(36, -88, 2)	54	1.29
5	L	10	(-30, 56, 6)	149	1.72
6	L	9, 46	(-46, 10,30)	410	1.67
7	L	9, 10	(-32, 40,32)	66	1.28
8	R	9, 10	(32, 40, 32)	79	1.35
9	R	8	(32, 26, 44)	8	1.04
10	L	8	(-24, 30,46)	16	1.08
11	R	6	(36, -4, 58)	23	1.1

^a^ Reported by xjView.

^b^ Left or right cerebral hemisphere.

^c^ Brodmann areas.

^d^ Montreal Neurological Institute coordinates for the peak voxel.

^e^ Number of voxels in each cluster.

^f^ Peak value of degree centrality.

### Test-retest reliability of voxel-wise degree centrality

**Fig 4:**
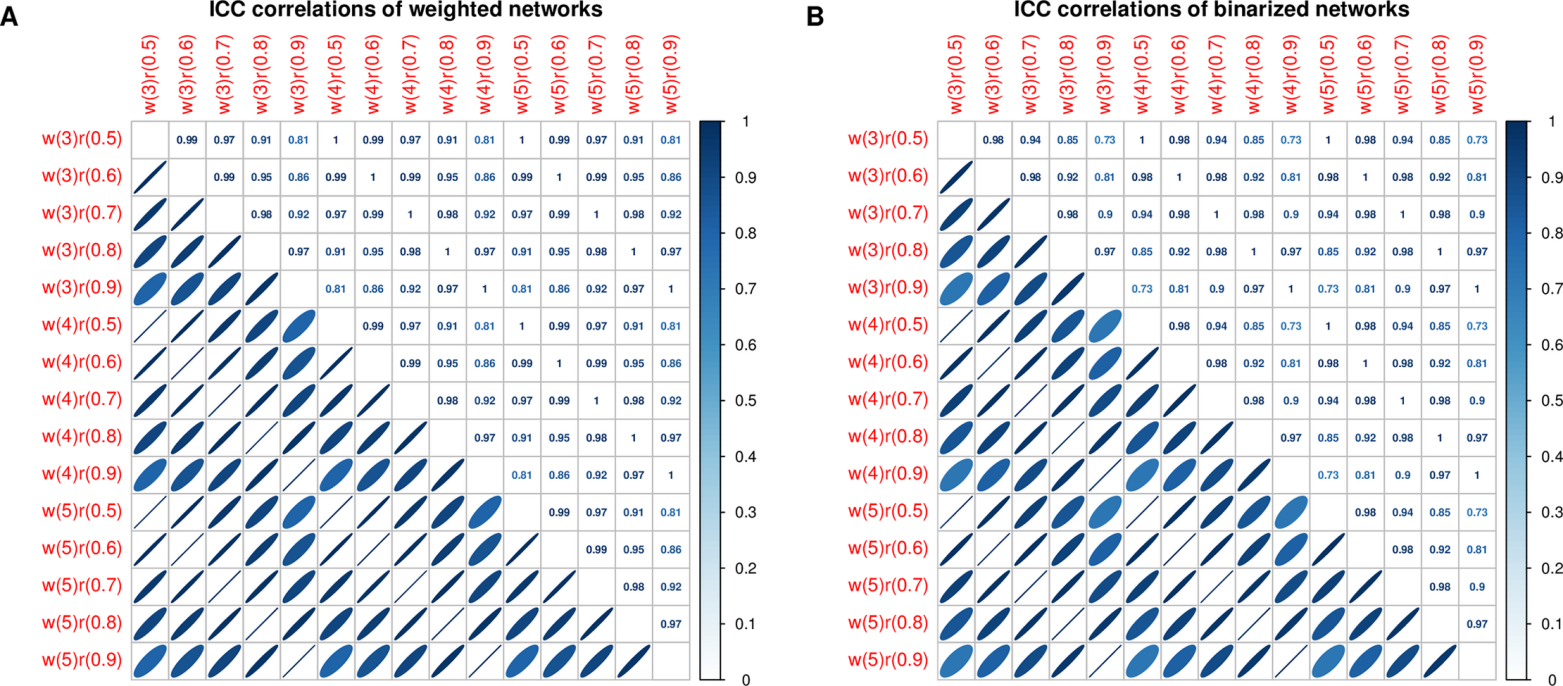
Correlations of degree centrality reliability among different networks. Subfigure A denotes the correlations of degree centrality reliability for weighted networks. Subfigure B denotes the correlations of degree centrality reliability for binarized networks. w is the wavelet scale, and r is the threshold of the network.

**Fig 5:**
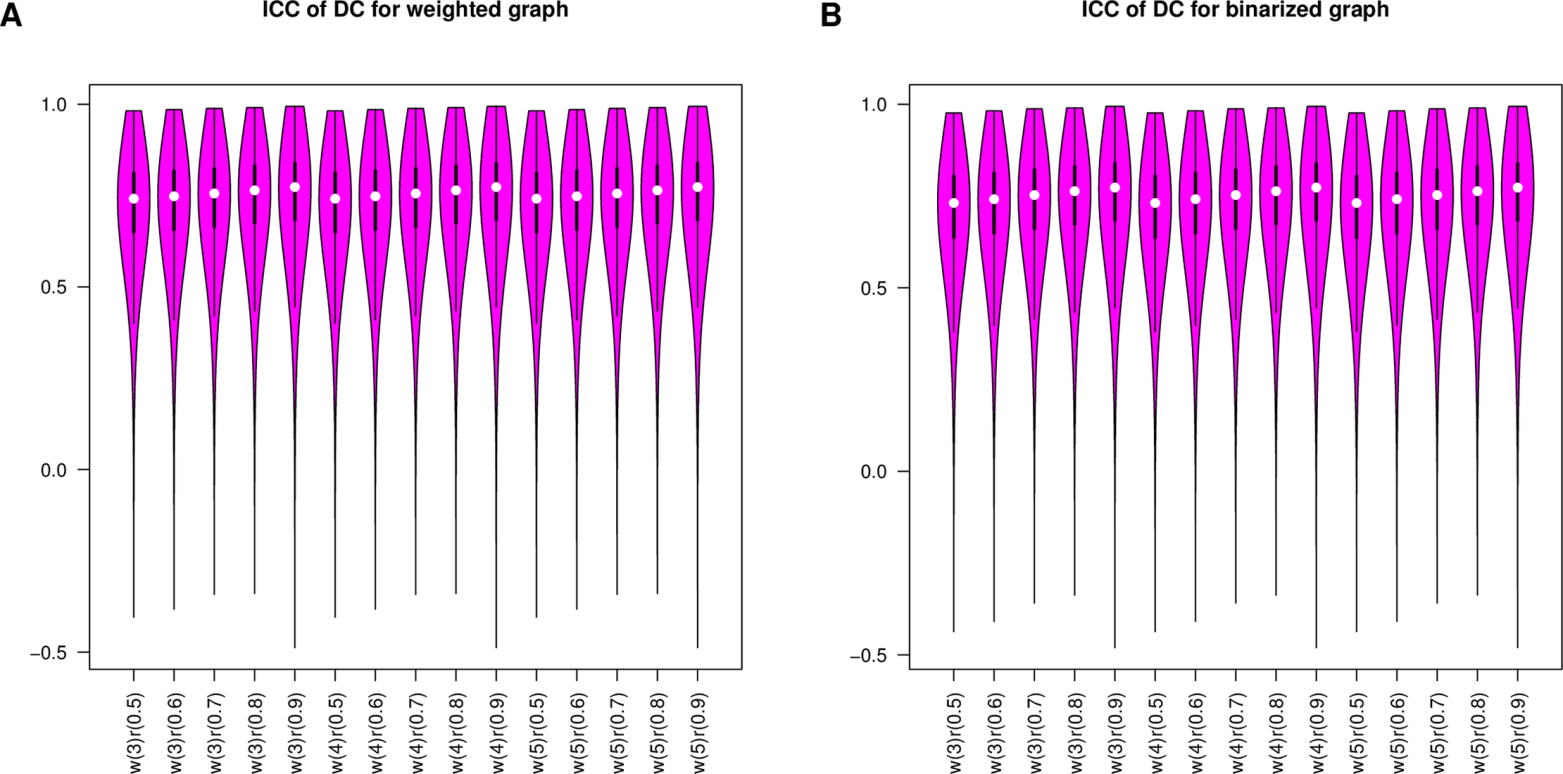
Violin plots of degree centrality reliability. Subfigure A denotes the violin plots of degree centrality reliability for weighted networks. Subfigure B denotes the violin plots of degree centrality reliability for binarized networks. w is the wavelet scale, and r is the threshold of the network.

**Fig 6:**
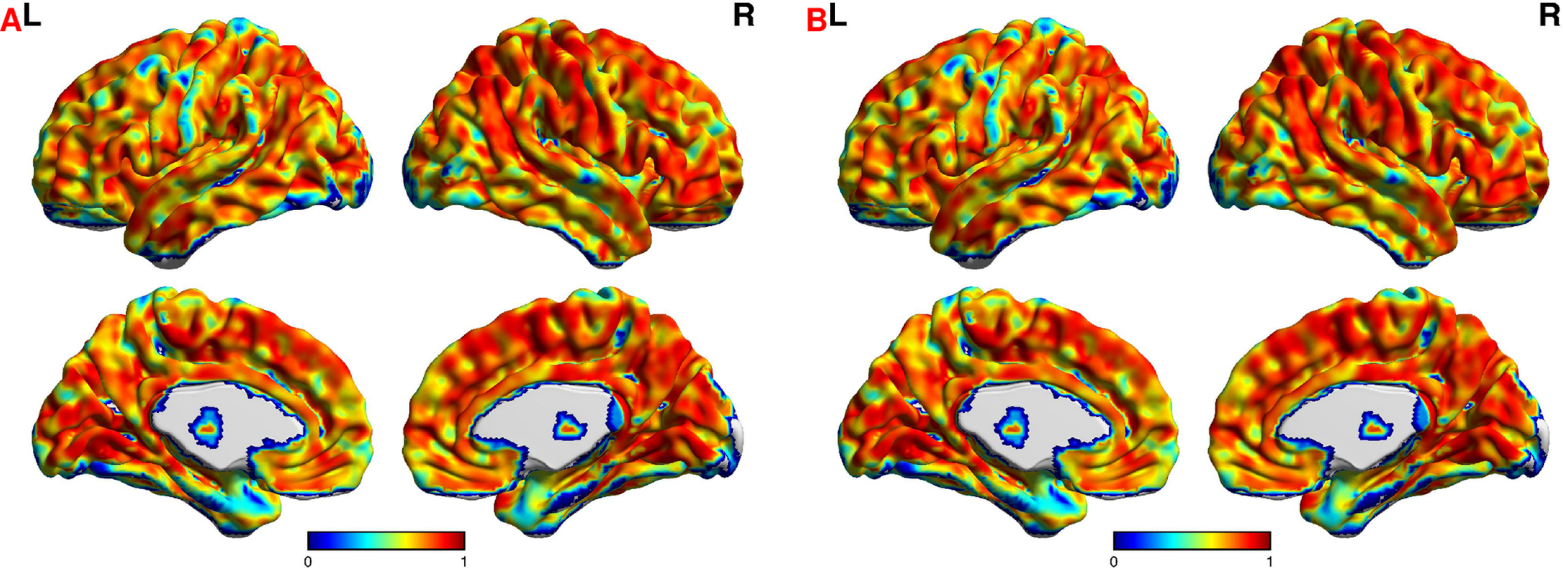
Mean reliability of degree centrality with a threshold of 0.8. Subfigure A denotes the mean reliability of degree centrality for weighted networks with a threshold of 0.8. Subfigure B denotes the mean reliability of degree centrality for binarized networks with a threshold of 0.8.

Table 6 show the results of three-way ANOVA for reliability of degree centrality. There are no effects of wavelet scale on the reliability of voxel-wise degree centrality. However, the network threshold and network type might affect the reliability of voxel-wise degree centrality. Fig 4 shows correlations of degree centrality reliability among different networks. Most of the correlation coefficients are beyond 0.7. Moreover, the reliability maps are significantly correlated with each other. Fig 5 shows violin plots for the reliability of degree centrality. Most of the ICC values exceed 0.6, which indicates high reliability. Table 7 shows the mean and standard deviation of reliability. Most of the mean values of ICCs exceed 0.7 with standard deviations of less than 0.14. High-to-excellent reliability of degree centrality can be observed in Fig 5 and Table 7. Fig 6 shows the maps of reliability with a network threshold of 0.8. The voxel-wise degree centrality exhibits high test-retest reliability across different networks.

**Table 6:** ANOVA of degree centrality reliability.

	sum square	mean square	F value	p-value
wavelet scales	0	0	0	1
threshold	641	160.2	8492.4	<10^−10^
network type	20	20.3	1076.2	<10^−10^

**Table 7:** Mean and standard deviation of ICC.

	r = 0.5	r = 0.6	r = 0.7	r = 0.8	r = 0.9
Binarized scale 3	0.71 ± 0.14	0.72 ± 0.14	0.73 ± 0.14	0.74 ± 0.14	0.74 ± 0.14
Binarized scale 4	0.71 ± 0.14	0.72 ± 0.14	0.73 ± 0.14	0.74 ± 0.14	0.74 ± 0.14
Binarized scale 5	0.71 ± 0.14	0.72 ± 0.14	0.73 ± 0.14	0.74 ± 0.14	0.74 ± 0.14
Weighted scale 3	0.72 ± 0.14	0.73 ± 0.14	0.73 ± 0.14	0.74 ± 0.14	0.74 ± 0.14
Weighted scale 4	0.72 ± 0.14	0.73 ± 0.14	0.73 ± 0.14	0.74 ± 0.14	0.74 ± 0.14
Weighted scale 5	0.72 ± 0.14	0.73 ± 0.14	0.73 ± 0.14	0.74 ± 0.14	0.74 ± 0.14

### Individual-level degree centrality of the voxel-wise networks

The repeated-measures ANOVA analysis revealed significant (p<0.05, FDR corrected) main effects and interaction effects of the three different factors (i.e., threshold, scale, type) on individual-level degree centrality. A significant main effect of network threshold was found in 91.62% of the whole-brain voxels. A significant main effect of wavelet scale was found in 97.16% of the whole-brain voxels. A significant main effect of network type was found in 81.84% of the whole-brain voxels. A significant threshold:scale interaction was found in 99.1% of the whole-brain voxels. A significant threshold:type interaction was found in 88.24% of the whole-brain voxels. A significant scale:type interaction was found in 96.12% of the whole brain voxels. A significant threshold:scale:type interaction was found in 98.55% of the whole-brain voxels.

## Discussion

In this study, we sought to map voxel-wise morphological connectivity at the single-subject level based on anatomical MRI. To this end, wavelet transform was applied on the VBM features. The morphological connectivity was obtained via the inter-voxel hierarchical wavelet features. The voxel-wise morphological networks exhibited good test-retest reliability across different wavelet scales, different network thresholds, and different network types. In addition, the voxel-wise morphological networks exhibited hub structures, most of which were located in the default mode network (DMN) area. Overall, the inter-voxel morphological relationships were successfully obtained for individuals through wavelet transform. The voxel-wise morphological connectivity might provide additional measures for studies of the human connectome.

Wavelet transform can capture the local and global features of individual MRI datasets in a multi-resolution manner. Thus, individual brain networks can be built through wavelet-based hierarchical features in a straightforward manner. Wavelet transform has been employed as an efficient tool for feature extraction and signal representation [17]. The components of wavelet transform might reflect the biological meanings of the original images. The low-frequency information decoded by wavelet transform might be beneficial in assisting in the diagnostic process of brain disorders [18]. Therefore, wavelet transform could provide rich information for disease classification and advance the analysis of neuroimaging data. Moreover, wavelet features can indicate inherent hierarchical structures, which could be used to probe multiscale information of brain imaging datasets. In contrast to previous studies, this study for the first time mapped the voxel-wise morphological networks, which correspond to multivariate measures. Furthermore, the results revealed that the wavelet scales had no effects on group-level degree centrality or its reliability, suggesting that wavelet transform could capture reliable features that could be beneficial to the brain connectome. However, the biological meanings of the morphological network remain unclear. Possible explanations can be drawn from the axon tension theory [13] or that morphologically connected brain regions may share similar structures for information transmission.

The hub regions, which are connected to more brain nodes than other regions, may reflect the functional or structural cores within the brain networks. In this study, we found certain brain regions that exhibited hub organization (i.e., the precuneus, the cingulate gyrus, the medial frontal gyrus, the superior frontal gyrus, and the superior temporal gyrus), most of which were located in the DMN area. DMN-related morphological hubs might reflect structural or functional connections. Pervious evidence has indicated structural cores located in brain regions of the posterior components of the DMN [21]. High global connectivity has been found in the DMN regions and cognitive control regions, suggesting the important roles of hub regions in information processing [30]. Moreover, the low percentage of network nodes with high degree centrality found in this study suggested that the morphological connectivity might exhibit scale-free organization. The human brain functional network has been shown to be regulated by highly connected cortical cores and exhibits power-law degree distribution [31]. Therefore, the brain hubs detected by morphological connectivity could be supplementary to conventional functional and structural cores. In summary, the morphological hubs might possess functional and structural meanings and might be related to brain disorders [32] and development [33].

The test-retest reliability is a key feature for evaluating novel neural metrics [34]. The active patterns (i.e., Amplitude of Low Frequency Fluctuations (ALFF), Regional Homogeneity (ReHo), entropy) of resting-state blood-oxygenation level-dependent (BOLD) signals were reliable at the voxel level [24, 27, 35]. Reliable voxel-wise measures of VBM were found using multicenter datasets [36]. In this paper, the voxel-wise degree centrality maps were derived from the VBM features. Thus, the proposed voxel-wise morphological networks should exhibit certain test-retest reliability. Reliable hub structures of voxel-wise morphological networks at the group level were found across the two scan sessions. High reliability of the voxel-wise morphological networks was confirmed by the test-retest analysis. The reproducible hubs suggested that wavelet-based morphological connectivity might be a reliable metric to probe voxel-wise brain connectivity. However, the network thresholds and network types might impact the test-retest reliability of voxel-wise networks. The results suggested that the network thresholds and network types should be carefully selected to verify the test-retest reliability of morphological networks. Nevertheless, high ICC values were obtained across different wavelet scales, network thresholds, and network types, implying that the morphological connectivity is a reliable measure for clinical applications.

Individual differences are a fundamental property of a novel neural metric. Previous individual connectivity was based on diffusion MRI or functional MRI, which are both sensitive to confounding artifacts [13]. Given the well-established scanning and preprocessing procedures, anatomical MRI could be beneficial for understanding individual variability. Notably, previous structural covariance networks based on group-level measures could not represent individual differences [37, 38]. Source-based morphometry (SBM) is a novel method based on group-level independent component analysis (ICA) of anatomical MRI data [39]. The method proposed in this study could be extended to individual-level SBM, as a wavelet-based 4D volume was obtained for each subject. Thus, individual differences of spatial sources based on SBM could be investigated using wavelet features. In this study, repeated-measures ANOVA revealed significant individual differences in the morphological connectivity. Significant main and interaction effects of the three factors (i.e., network threshold, wavelet scale, network type) were found throughout the whole brain, suggesting that the network factors could influence individual differences. Therefore, network factors should be taken into account when studying individual differences in the proposed morphological connectivity. To the best of our knowledge, this is the first study to map individual voxel-wise morphological connectivity based on anatomical MRI. The proposed morphological connectivity could advance the statistical analysis of individual differences and machine learning-based diagnostic models.

This study is characterized by several advantages. First, the voxel-wise morphological network was investigated for single subjects in a wavelet-based framework. Evaluating the voxel-wise morphological connectivity for single subjects might be beneficial for investigating individual differences. Second, the wavelet transform could yield hierarchical features (i.e., global features, local features). Thus, the wavelet-based morphological connectivity could capture multi-resolution information. Third, the voxel-wise morphological connectivity detected reliable hub structures. The reliable hub structures identified by the framework might reflect the functional or structural basis of voxel-wise morphological connectivity. Fourth, the voxel-wise morphological estimators exhibited good test-retest reliability. Reliability is an essential feature for novel neural metrics. The reliable voxel-wise morphological estimators implied their potential values in clinical applications. In summary, the proposed morphological connectivity method was reliable across different network levels and could advance the study of individual variability.

One limitation of this study is that the biological meanings of the proposed voxel-wise morphological connectivity feature could not be elucidated. Although reliable voxel-wise morphological connectivity was discovered by wavelet-based features, supplemental experiments are still required to test the hypothesis that morphological connectivity can reflect structural and functional connectivity. Moreover, there are numerous similarity and dissimilarity metrics (i.e., KL divergence, correlative features) that can be applied to measure the morphological connectivity in additional studies. Another limitation of this study is the lack of physiological signals for references. The morphological similarity-based results could be interpreted with supplementary physiological measures (i.e., electroencephalography (EEG), magnetoencephalography (MEG)) in future studies. The third limitation of this study is the relatively small sample size of participants. The results should be verified using different scanners and different populations in subsequent studies. Together, there are still many challenges that must be overcome in the study of voxel-wise morphological networks. Additional physiological measures might help interpret the novel individual morphological networks.

## Conclusion

This paper proposed a voxel-wise wavelet-based similarity measure to evaluate morphological connectivity at the single-subject level based on structural MRI. The morphological networks were reliable across different wavelet scales, network thresholds, and network types. The voxel-wise morphological connectivity exhibited excellent reliability and reflected individual differences. In summary, the voxel-wise wavelet-based similarity could probe individual morphological connectivity and could be beneficial for investigating the brain morphological connectome.

## References

[1] Ashburner J, Friston KJ. Voxel-based morphometry—the methods. NeuroImage. 2000;11(6 Pt 1):805–21. 10.1006/nimg.2000.0582 . 10860804

[2] Maguire EA, Gadian DG, Johnsrude IS, Good CD, Ashburner J, Frackowiak RS, et al Navigation-related structural change in the hippocampi of taxi drivers. Proc Natl Acad Sci U S A. 2000;97(8):4398–403. 10.1073/pnas.070039597 10716738 PMC18253

[3] Ecker C, Rocha-Rego V, Johnston P, Mourao-Miranda J, Marquand A, Daly EM, et al Investigating the predictive value of whole-brain structural MR scans in autism: a pattern classification approach. NeuroImage. 2010;49:44–56. 10.1016/j.neuroimage.2009.08.024 . 19683584

[4] Kawasaki Y, Suzuki M, Kherif F, Takahashi T, Zhou SY, Nakamura K, et al Multivariate voxel-based morphometry successfully differentiates schizophrenia patients from healthy controls. NeuroImage. 2007;34(1):235–42. 10.1016/j.neuroimage.2006.08.018 . 17045492

[5] Pantazatos SP. Prediction of individual season of birth using MRI. NeuroImage. 2013;88:61–8. 10.1016/j.neuroimage.2013.11.011 . 24246490 PMC4545475

[6] Bullmore E, Sporns O. Complex brain networks: graph theoretical analysis of structural and functional systems. Nat Rev Neurosci. 2009;10:186–98. 10.1038/nrn2575 . 19190637

[7] Rubinov M, Sporns O. Complex network measures of brain connectivity: uses and interpretations. NeuroImage. 2010;52(3):1059–69. 10.1016/j.neuroimage.2009.10.003 . 19819337

[8] Achard S, Salvador R, Whitcher B, Suckling J, Bullmore E. A resilient, low-frequency, small-world human brain functional network with highly connected association cortical hubs. J Neurosci. 2006;26:63–72. 10.1523/JNEUROSCI.3874-05.2006 . 16399673 PMC6674299

[9] van den Heuvel MP, Sporns O, Heuvel Mvd. Rich-club organization of the human connectome. J Neurosci. 2011;31(44):15775–86. 10.1523/JNEUROSCI.3539-11.2011 . 22049421 PMC6623027

[10] Ciuciu P, Varoquaux G, Abry P, Sadaghiani S, Kleinschmidt a. Scale-Free and Multifractal Time Dynamics of fMRI Signals during Rest and Task. Front Physiol. 2012;3:186 10.3389/fphys.2012.00186 . 22715328 PMC3375626

[11] Yan C-G, Craddock RC, Zuo X-N, Zang Y-F, Milham MP. Standardizing the intrinsic brain: towards robust measurement of inter-individual variation in 1000 functional connectomes. NeuroImage. 2013;80:246–62. 10.1016/j.neuroimage.2013.04.081 . 23631983 PMC4074397

[12] Baum GL, Roalf DR, Cook PA, Ciric R, Rosen AFG, Xia C, et al The impact of in-scanner head motion on structural connectivity derived from diffusion MRI. NeuroImage. 2018;173:275–86. 10.1016/j.neuroimage.2018.02.041 ; PubMed Central PMCID: PMCPMC5911236. 29486323 PMC5911236

[13] Kong X-Z, Wang X, Huang L, Pu Y, Yang Z, Dang X, et al Measuring individual morphological relationship of cortical regions. Journal of neuroscience methods. 2014;237:103–7. 10.1016/j.jneumeth.2014.09.003 . 25220868

[14] Wang H, Jin X, Zhang Y, Wang J. Single-subject morphological brain networks: connectivity mapping, topological characterization and test-retest reliability. Brain Behav. 2016;6(4):e00448 10.1002/brb3.448 ; PubMed Central PMCID: PMCPMC4782249. 27088054 PMC4782249

[15] Kong XZ, Liu Z, Huang L, Wang X, Yang Z, Zhou G, et al Mapping Individual Brain Networks Using Statistical Similarity in Regional Morphology from MRI. PloS one. 2015;10(11):e0141840 10.1371/journal.pone.0141840 ; PubMed Central PMCID: PMCPMC4633111. 26536598 PMC4633111

[16] Tijms BM, Serie P, Willshaw DJ, Lawrie SM. Similarity-Based Extraction of Individual Networks from Gray Matter MRI Scans. Cerebral cortex. 2012;22:1530–41. 10.1093/cercor/bhr221 21878484

[17] Canales-Rodriguez EJ, Radua J, Pomarol-Clotet E, Sarro S, Aleman-Gomez Y, Iturria-Medina Y, et al Statistical analysis of brain tissue images in the wavelet domain: wavelet-based morphometry. NeuroImage. 2013;72:214–26. 10.1016/j.neuroimage.2013.01.058 . 23384522

[18] Hackmack K, Paul F, Weygandt M, Allefeld C, Haynes J-D. Multi-scale classification of disease using structural MRI and wavelet transform. NeuroImage. 2012;62:48–58. 10.1016/j.neuroimage.2012.05.022 . 22609452

[19] Landman BA, Huang AJ, Gifford A, Vikram DS, Lim IAL, Farrell JAD, et al Multi-parametric neuroimaging reproducibility: A 3-T resource study. NeuroImage. 2011;54:2854–66. 10.1016/j.neuroimage.2010.11.047 . 21094686 PMC3020263

[20] Zuo X-N, Ehmke R, Mennes M, Imperati D, Castellanos FX, Sporns O, et al Network centrality in the human functional connectome. Cerebral cortex. 2012;22(8):1862–75. 10.1093/cercor/bhr269 . 21968567

[21] Hagmann P, Cammoun L, Gigandet X, Meuli R, Honey CJ, Wedeen VJ, et al Mapping the structural core of human cerebral cortex. PLoS Biol. 2008;6(7):e159 10.1371/journal.pbio.0060159 . 18597554 PMC2443193

[22] Lohmann G, Margulies DS, Horstmann A, Pleger B, Lepsien J, Goldhahn D, et al Eigenvector centrality mapping for analyzing connectivity patterns in FMRI data of the human brain. PloS one. 2010;5:e10232 10.1371/journal.pone.0010232 . 20436911 PMC2860504

[23] Yan C-G, Zang Y-F. DPARSF: A MATLAB Toolbox for "Pipeline" Data Analysis of Resting-State fMRI. Front Syst Neurosci. 2010;4:13 10.3389/fnsys.2010.00013 ; PubMed Central PMCID: PMC2889691. 20577591 PMC2889691

[24] Liao X-H, Xia M-R, Xu T, Dai Z-J, Cao X-Y, Niu H-J, et al Functional brain hubs and their test-retest reliability: A multiband resting-state functional MRI study. NeuroImage. 2013;83:969–82. 10.1016/j.neuroimage.2013.07.058 . 23899725

[25] Dai Z, Yan C, Li K, Wang Z, Wang J, Cao M, et al Identifying and Mapping Connectivity Patterns of Brain Network Hubs in Alzheimer ‘ s Disease. Cerebral cortex. 2014;bhu246:1–20. 10.1093/cercor/bhu246 25331602

[26] Xia M, Wang J, He Y. BrainNet Viewer: A Network Visualization Tool for Human Brain Connectomics. PloS one. 2013;8:e68910 10.1371/journal.pone.0068910 23861951 PMC3701683

[27] Zuo X-N, Di Martino A, Kelly C, Shehzad ZE, Gee DG, Klein DF, et al The oscillating brain: complex and reliable. NeuroImage. 2010;49(2):1432–45. 10.1016/j.neuroimage.2009.09.037 . 19782143 PMC2856476

[28] Caceres A, Hall DL, Zelaya FO, Williams SCR, Mehta MA. Measuring fMRI reliability with the intra-class correlation coefficient. NeuroImage. 2009;45:758–68. 10.1016/j.neuroimage.2008.12.035 19166942

[29] Wang X, Jiao Y, Tang T, Wang H, Lu Z. Investigating univariate temporal patterns for intrinsic connectivity networks based on complexity and low-frequency oscillation: a test-retest reliability study. Neuroscience. 2013;254:404–26. 10.1016/j.neuroscience.2013.09.009 . 24042040

[30] Cole MW, Pathak S, Schneider W. Identifying the brain's most globally connected regions. NeuroImage. 2010;49:3132–48. 10.1016/j.neuroimage.2009.11.001 . 19909818

[31] van den Heuvel MP, Stam CJ, Boersma M, Hulshoff Pol HE. Small-world and scale-free organization of voxel-based resting-state functional connectivity in the human brain. NeuroImage. 2008;43:528–39. 10.1016/j.neuroimage.2008.08.010 . 18786642

[32] Buckner RL, Sepulcre J, Talukdar T, Krienen FM, Liu H, Hedden T, et al Cortical hubs revealed by intrinsic functional connectivity: mapping, assessment of stability, and relation to Alzheimer's disease. J Neurosci. 2009;29(6):1860–73. 10.1523/JNEUROSCI.5062-08.2009 . 19211893 PMC2750039

[33] Cao M, Wang J-H, Dai Z-J, Cao X-Y, Jiang L-L, Fan F-M, et al Topological organization of the human brain functional connectome across the lifespan. Dev Cogn Neurosci. 2014;7:76–93. 10.1016/j.dcn.2013.11.004 . 24333927 PMC6987957

[34] Zuo X-N, Xing X-X. Test-retest reliabilities of resting-state FMRI measurements in human brain functional connectomics: A systems neuroscience perspective. Neuroscience and Biobehavioral Reviews. 2014;45:100–18. 10.1016/j.neubiorev.2014.05.009 24875392

[35] Zuo X-N, Xu T, Jiang L, Yang Z, Cao X-Y, He Y, et al Toward reliable characterization of functional homogeneity in the human brain: Preprocessing, scan duration, imaging resolution and computational space. NeuroImage. 2013;65:374–86. 10.1016/j.neuroimage.2012.10.017 . 23085497 PMC3609711

[36] Schnack HG, van Haren NE, Brouwer RM, van Baal GC, Picchioni M, Weisbrod M, et al Mapping reliability in multicenter MRI: voxel-based morphometry and cortical thickness. Hum Brain Mapp. 2010;31(12):1967–82. 10.1002/hbm.20991 . 21086550 PMC6870904

[37] Brandon A. Zielinski EDG, Juan Zhou, Seeley WW. Network-level structural covariance in the developing brain. Proc Natl Acad Sci U S A. 2010;107(42):18191–6. 10.1073/pnas.1003109107 20921389 PMC2964249

[38] Guo X, Wang Y, Guo T, Chen K, Zhang J, Li K, et al Structural Covariance Networks Across Healthy Young Adults and Their Consistency. Journal of magnetic resonance imaging: JMRI. 2014 10.1002/jmri.24780 25327998

[39] Xu L, Groth KM, Pearlson G, Schretlen DJ, Calhoun VD. Source-based morphometry: the use of independent component analysis to identify gray matter differences with application to schizophrenia. Hum Brain Mapp. 2009;30(3):711–24. 10.1002/hbm.20540 ; PubMed Central PMCID: PMCPMC2751641. 18266214 PMC2751641

